# Human chorionic gonadotropin promotes murine Treg cells and restricts pregnancy-harmful proinflammatory Th17 responses

**DOI:** 10.3389/fimmu.2022.989247

**Published:** 2022-09-20

**Authors:** Lea S. Lentz, Annika J. Stutz, Nicole Meyer, Kristin Schubert, Isabel Karkossa, Martin von Bergen, Ana C. Zenclussen, Anne Schumacher

**Affiliations:** ^1^ Experimental Obstetrics and Gynecology, Medical Faculty, Health Campus Immunology, Infectilogy and Inflammation (GC-I^3^), Otto-von-Guericke University, Magdeburg, Germany; ^2^ Department of Environmental Immunology, Helmholtz Centre for Environmental Research, Leipzig, Germany; ^3^ Department of Molecular Systems Biology, Helmholtz Centre for Environmental Research, Leipzig, Germany; ^4^ Faculty of Life Sciences, Institute of Biochemistry, University of Leipzig, Leipzig, Germany; ^5^ German Centre for Integrative Biodiversity Research (iDiv) Halle-Jena-Leipzig, Leipzig, Germany

**Keywords:** human chorionic gonadotropin, regulatory T cells, type 17 T helper cells, T cell equilibrium, fetal tolerance, fetal and placental growth, uterine vascular bed, pregnancy

## Abstract

An equilibrium between proinflammatory and anti-inflammatory immune responses is essential for maternal tolerance of the fetus throughout gestation. To study the participation of fetal tissue-derived factors in this delicate immune balance, we analyzed the effects of human chorionic gonadotropin (hCG) on murine Treg cells and Th17 cells *in vitro*, and on pregnancy outcomes, fetal and placental growth, blood flow velocities and remodeling of the uterine vascular bed *in vivo*. Compared with untreated CD4^+^CD25^+^ T cells, hCG increased the frequency of Treg cells upon activation of the LH/CG receptor. hCG, with the involvement of IL-2, also interfered with induced differentiation of CD4^+^ T cells into proinflammatory Th17 cells. In already differentiated Th17 cells, hCG induced an anti-inflammatory profile. Transfer of proinflammatory Th17 cells into healthy pregnant mice promoted fetal rejection, impaired fetal growth and resulted in insufficient remodeling of uterine spiral arteries, and abnormal flow velocities. Our works show that proinflammatory Th17 cells have a negative influence on pregnancy that can be partly avoided by *in vitro* re-programming of proinflammatory Th17 cells with hCG.

## Introduction

Successful pregnancy requires coordination of fetal–maternal communication to enable survival of the semi-allogeneic fetus within the maternal womb. In particular, fetal tissue-derived factors enable fine regulation of maternal innate and adaptive immune cell populations (1, 2). Within this immunological network, T cells function as key regulators. Several Th cell subsets of either proinflammatory or anti-inflammatory nature are important determinants of fetal fate (3). Their peripheral and local frequencies, functionalities and relative frequencies are all important for the establishment and progression of pregnancy. Notably, the balance between Treg cells and Th17 cells was postulated to be pivotal for early and late pregnancy outcomes (4, 5).

Treg cells are a Th subset with great potential to regulate the phenotype and functionality of other immune-cell populations. They are involved in the prevention of autoimmune diseases (6), allograft rejection (7), graft-versus-host disease (8) and antitumor reactions (9). Treg cells are characterized by expression of the transcription factor forkhead box protein P3 (FOXP3), the immune-checkpoint inhibitors cytotoxic T-lymphocyte protein 4 (CTLA-4) and programmed cell death protein 1 (PD-1), as well as by the production of the immunosuppressive cytokines IL-10, IL-35 and TGF-β (10). Th17 cells are important for defense against extracellular bacterial and fungal pathogens and are negatively associated with tissue inflammation and organ-specific autoimmunity (11). Characteristic features of Th17 cells include expression of the transcription factor retinoic acid receptor-related orphan receptor γt (RORγt), secretion of cytokines IL-17, IL-21 and IL-22, and production of GM-CSF (12). Both Th subsets exhibit considerable plasticity and can be reprogrammed into other Th subsets, such as Th1 and Th2 (13, 14). Moreover, under certain conditions, Treg cells transdifferentiate into Th17 cells, and vice versa (13, 14).

Results from human and murine studies have demonstrated associations between deregulation of the Treg : Th17 ratio and the spontaneous termination of pregnancy. Elevation of Th17 levels and reduction of Treg levels in human peripheral blood and in the decidua have been detected in patients with unexplained recurrent spontaneous abortion (URSA), compared with the levels in women with normal pregnancies, or in fertile nonpregnant women (15–20). Notably, intravenous application of IgG or a combined immunotherapy of paternal mononuclear cells and vitamin D3 in patients with URSA restores the Treg : Th17 balance (21, 22) and seems to be a promising therapeutic intervention for prevention of miscarriage. However, no direct proof yet exists for improvement of pregnancy outcomes in patients with URSA through targeting of Treg:Th17. In mice, intraperitoneal injection of recombinant IL-17, the main cytokine of Th17 cells, induces fetal loss in otherwise healthy pregnant females, whereas Ab-mediated blockage of IL-17 rescues fetuses in abortion-prone females (23). The higher expression of IL-17 and RORγt at abortion sites (sites of implantation followed by spontaneous termination) than at apparently normal implantation sites (23) suggests an association between Th17 levels and pregnancy failure in mice similar to that observed in humans. In addition, in humans, disturbed Treg : Th17 ratios have been reported in patients suffering from pre-eclampsia (24–27), a pregnancy disorder characterized by the onset of high blood pressure and proteinuria. Pre-eclampsia is often caused by systemic chronic inflammation and oxidative stress (28), and is sometimes accompanied by intrauterine growth restriction (IUGR) (29). Mechanisms contributing to IUGR include shallow trophoblastic invasion into the uterine spiral arteries (uSAs), impeding remodeling of the vascular bed and leading to uterine hypoxia (30), reduction of the fetal blood supply, and growth restriction because of fetal undernourishment. Notably, failure of maternal immune tolerance has been reported to be causative for uterovascular resistance in IUGR (31).

Evidence suggests that the Th17 cell pool is not *per se* detrimental to pregnancy, as it consists of harmful proinflammatory and beneficial regulatory subsets and the relative frequency of the subsets may vary depending on intrinsic and extrinsic factors. In a study of the decidua from women with spontaneous or elective termination of pregnancy, only Th17 cells coproducing IL-17 and IFN-γ were identified in patients with URSA, whereas most Th17 cells coproduced IL-17 and IL-4 in women with healthy pregnancies (32). However, the factors that determine differentiation of Th17 cells during pregnancy to pathogenic or tolerogenic phenotypes have not yet been defined.

One of the most prominent products of human pregnancy is the placenta-derived hormone human chorionic gonadotropin (hCG), which is already detectable 10 days after fertilization in the maternal circulation (33), increasing dramatically during the first weeks of pregnancy to peak in weeks 9–10 and subsequently decline (34). Although hCG is a primate-specific hormone, its functionality can also be studied in the murine system, because its receptor, the luteinizing hormone/chorionic gonadotropin receptor (LH/CGR), is highly conserved, and hCG binds efficiently to the murine LH/CGR (35). In addition to pregnancy-preserving biological activities, hCG has immunomodulatory properties, and is considered to be one of the main regulatory factors for induction of fetal tolerance (36, 37). Both urine-derived hCG (uhCG) and recombinant hCG (rhCG) are used clinically to improve implantation and live-birth rates in patients undergoing *in vitro* fertilization (IVF), although these two forms of hCG differ in their efficacies (38, 39). uhCG contains all naturally occurring hCG isoforms that are present during pregnancy, whereas rhCG consists of a single hCG isoform. uhCG preparations also contain notable levels of non-hCG proteins (up to 30%) and have high batch-to-batch variability, whereas rhCG preparations are >99% pure, with high batch-to-batch consistency and can be used at much lower concentrations than uhCG (38). Intrauterine administration of hCG-activated autologous PBMCs has been shown to improve implantation and pregnancy rates in patients with repeated implantation failure (40–42), possibly *via* promotion of trophoblastic invasion (43). Additionally, hCG application before and after embryo transfer has been shown to reduce expression of proinflammatory IL-17A in mononuclear cells and to increase numbers of peripheral Treg cells (44). Nonetheless, there remains a substantial gap in our understanding of how hCG affects the various immune-cell populations.

In this study, we aimed to understand how hCG affects Treg and Th17 cell subpopulations *in vitro* and *in vivo*. In particular, we analyzed the effects of uhCG and rhCG on Treg cells, on Th17 generation and on Th17 plasticity *in vitro*. We further studied hCG-dependent effects of Th17 cell transfer in a murine pregnancy model *in vivo*.

## Results

### rhCG and uhCG act on CD4^+^CD25^+^ T cells through the LH/CGR to increase the proportions of Treg cells

**Figure 1:**
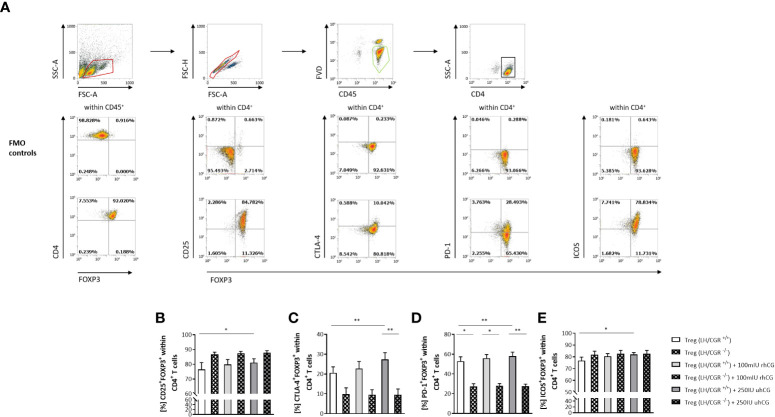
rhCG and uhCG stimulated formation of Treg cells through the LH/CGR *in vitro.* CD4^+^CD25^+^ T cells were isolated by magnetic-activated cell sorting (MACS) and cultured for 24 h in the presence of 250 IU/ml uhCG or 100 mIU/ml rhCG, or absence of hCG as controls. Following culture, the frequencies of CD4^+^FOXP3^+^ Treg cells expressing CD25, CTLA-4, PD-1 or ICOS were determined by flow cytometry. **(A)** Representative dot plots showing the gating strategy and flow-cytometric results of control FMO staining and full staining for all marker molecules except for the lineage markers CD4 and CD45. **(B–E)** Graphs showing the percentages of CD25^+^FOXP3^+^, CTLA-4^+^FOXP3^+^, PD-1^+^FOXP3^+^ and ICOS^+^FOXP3^+^ cells within all CD45^+^CD4^+^ T cells. CD4^+^CD25^+^ T cells from WT (LH/CGR^+/+^;*n* = 6) or LH/CGR-deficient (LH/CGR^-/-^; *n* = 4) female mice were included in the assays. All assays were run in duplicate. Data are presented as the mean plus standard error of the mean (S.E.M.). Statistical analysis among LH/CGR^+/+^ or LH/CGR^-/-^ groups was performed using the Friedman test followed by Dunn’s multiple-comparison test. For comparisons between LH/CGR^+/+^ and LH/CGR^-/-^ groups, the Kruskal–Wallis test followed by Dunn’s multiple-comparison test was used. * indicates *p <*0.05; ** indicates *p <*0.01. Following, means plus S.E.M. are provided for significant differences between LH/CGR^+/+^ plus uhCG vs LH/CGR^+/+^ w/o hCG for CD25: 81.10 ± 2.606 vs 76.56 ± 4.575; CTLA-4: 27.37 ± 3.390 vs 20.60 ± 3.044; PD-1: 58.00 ± 4.020 vs 52.85 ± 4.442; ICOS: 81.95 ± 1.665 vs 76.75 ± 3.023 and LH/CGR^+/+^ plus uhCG vs LH/CGR^-/-^ plus uhCG for CTLA-4: 27.37 ± 3.390 vs 9.554 ± 2.877; LH/CGR^+/+^ vs LH/CGR^-/-^ for PD-1 52.85 ± 4.442 vs 27.39 ± 2.699 (w/o hCG); 55.94 ± 3.735 vs 27.99 ± 2.287 (rhCG); 58.00 ± 4.020 vs 27.80 ± 1.897 (uhCG). LH/CGR, luteinizing hormone/chorionic gonadotropin receptor; rhCG, recombinant hCG; uhCG, urine-derived hCG.

The ratio between the numbers of anti-inflammatory Treg cells and proinflammatory Th17 cells has been proposed to be decisive for pregnancy success. We therefore studied the effects of hCG on the frequencies of Treg cells expressing various surface molecules, and the involvement of the LH/CGR. CD4^+^CD25^+^ T cells were isolated from WT or LH/CGR-deficient virgin female mice, and were exposed to 100 mIU/ml rhCG or 250 IU/ml uhCG. Baseline proportions of CD4^+^CD25^+^ T cells included >90% FOXP3^+^ cells (**Figure 1A**). After exposure, the frequencies of viable CD45^+^CD4^+^ cells expressing CD25, CTLA-4, PD-1 and inducible T-cell costimulator (ICOS) in addition to the Treg marker FOXP3 (as well as the MFI of these markers within CD4^+^FOXP3^+^ cells) were assessed by flow cytometry. The gating strategy and representative dot plots for all markers analyzed (except lineage markers), along with the corresponding fluorescence minus one (FMO) controls, are shown in **Figure 1A**. Treatment of cells from WT mice with uhCG, but not with rhCG, resulted in significant increases in the frequencies of CD4^+^ cells co-expressing FOXP3^+^ and CD25, CTLA-4, PD-1 or ICOS (**Figures 1B–E**). MFI analyses did not demonstrate any effect of uhCG or rhCG on marker expression (**Supplementary Figure 1**). Notably, compared with the WT, CD4^+^ cells from LH/CGR-deficient mice included significantly lower proportions of cells co-expressing FOXP3^+^ and CTLA-4 or PD-1 (**Figures 1C, D**), and had lower ICOS expression (**Supplementary Figure 1D**). The cells from LH/CGR-deficient mice did not show any hCG-mediated Treg cell augmentation (**Figures 1B–E**), and expressed lower levels of CTLA-4, PD-1 and ICOS than WT cells in response to uhCG treatment (**Supplementary Figures 1B–D**). Secretion from WT-Treg cells of TGF-β, but not of IL-10 or IL-35, was significantly higher after rhCG treatment (but not after uhCG treatment) than from untreated cells (**Table 1**). Cytokine secretion from LH/CGR-deficient Treg cells was not significantly different to that from WT cells (**Table 1**). These findings suggest a promoting effect of hCG on Treg cell formation through the LH/CGR.

**Table 1:** Effects of recombinant hCG (rhCG) and urine-derived hCG (uhCG) on cytokine secretion by wild-type (LH/CGR^+/+^) and LH/CGR-deficient (LH/CGR^-/-^) Treg cells.

Cell type and treatment	Levels of cytokines in culture supernatants (pg/ml)		
	IL-10	IL-35	TGF-β
Treg (LH/CGR^+/+^)	24.80 ± 7.14	0.14 ± 0.06	929.1 ± 29.98
Treg (LH/CGR^-/-^)	28.04 ± 0.42	0.09 ± 0.02	970.0 ± 6.27
Treg (LH/CGR^+/+^) + 100 mIU/ml rhCG	39.65 ± 12.53	0.08 ± 0.03	954.4 ± 35.01*
Treg (LH/CGR^-/-^) + 100 mIU/ml rhCG	27.42 ± 3.87	0.09 ± 0.02	952.2 ± 20.51
Treg (LH/CGR^+/+^) + 250 IU/ml uhCG	38.49 ± 12.17	0.07 ± 0.03	938.8 ± 50.40
Treg (LH/CGR^-/-^) + 250 IU/ml uhCG	31.86 ± 1.69	0.11 ± 0.01	951.4 ± 5.58

Data are displayed as the mean plus standard error of the mean. Statistical analysis used the Friedman test followed by Dunn’s multiple-comparison test. * indicates p <0.05 vs Treg (LH/CGR^+/+^).

### rhCG and uhCG interfere with Th17 differentiation *in vitro*

**Figure 2:**
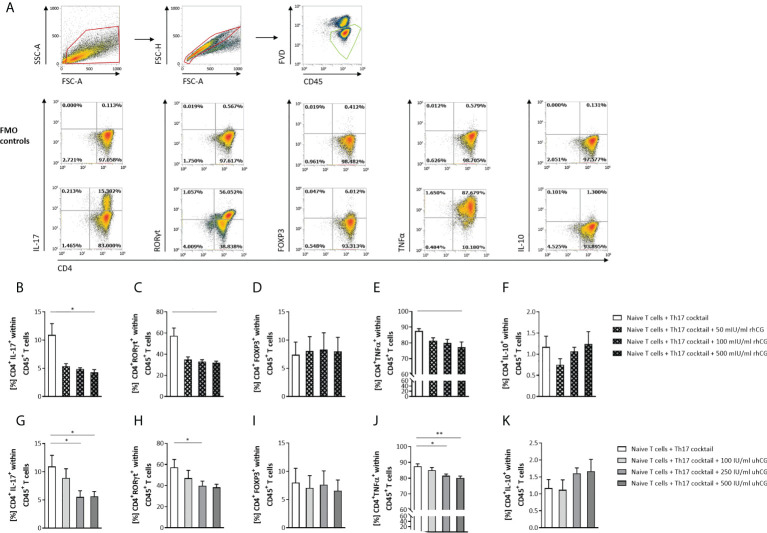
rhCG and uhCG interfered with Th17 differentiation *in vitro.* Naïve CD4^+^ T cells were isolated by magnetic-activated cell sorting (MACS) and polarized into Th17 cells under Th17-differentiating conditions for 3 days *in vitro*, in the presence of various concentrations of uhCG (100 IU/ml, 250 IU/ml and 500 IU/ml) or rhCG (50 mIU/ml, 100 mIU/ml and 500 mIU/ml), or absence of hCG as controls. Percentages of different T cell subsets as well as intracellular cytokine production were determined by flow cytometry. **(A)** Representative dot plots showing the gating strategy and flow-cytometric results of control FMO staining and full staining for all marker molecules except for the lineage markers CD4 and CD45. **(B–K)** Graphs showing the percentages of CD4^+^IL-17^+^, CD4^+^RORγt^+^, CD4^+^FOXP3^+^, CD4^+^TNFα^+^ and CD4^+^IL-10^+^ cells within CD45^+^ T cells in the rhCG **(B–F)** or uhCG **(G–K)** treatment groups. Naïve T cells from WT (*n* = 4) females mice were included in the assays. All assays were run in duplicate. Data are presented as the mean plus standard error of the mean (S.E.M.). Statistical analysis among groups was performed using the Friedman test followed by Dunn’s multiple-comparison test. Following, means plus S.E.M. are provided for significant differences between groups for CD4^+^IL-17^+^: 10.93 ± 1.989 vs 4.267 ± 0.5118 (w/o hCG vs 500mIU rhCG), 10.93 ± 1.989 vs 5.543 ± 1.069 (w/o hCG vs 250IU uhCG), 10.93 ± 1.989 vs 5.638 ± 0.8683 (w/o hCG vs 500IU uhCG); for CD4^+^RORγt^+^: 57.31 ± 7.485 vs 32.00 ± 1.380 (w/o hCG vs 500mIU rhCG), 57.31 ± 7.485 vs 39.80 ± 4.419 (w/o hCG vs 250IU uhCG); for CD4^+^TNFα^+^: 87.55 ± 1.541 vs 77.22 ± 3.392 (w/o hCG vs 500mIU rhCG), 87.55 ± 1.541 vs 81.59 ± 1.114 (w/o hCG vs 250IU uhCG), 87.55 ± 1.541 vs 80.12 ± 1.243 (w/o hCG vs 500IU uhCG). * indicates *p <*0.05; ** indicates *p <*0.01. rhCG, recombinant hCG; uhCG, urine-derived hCG.

**Figure 3:**
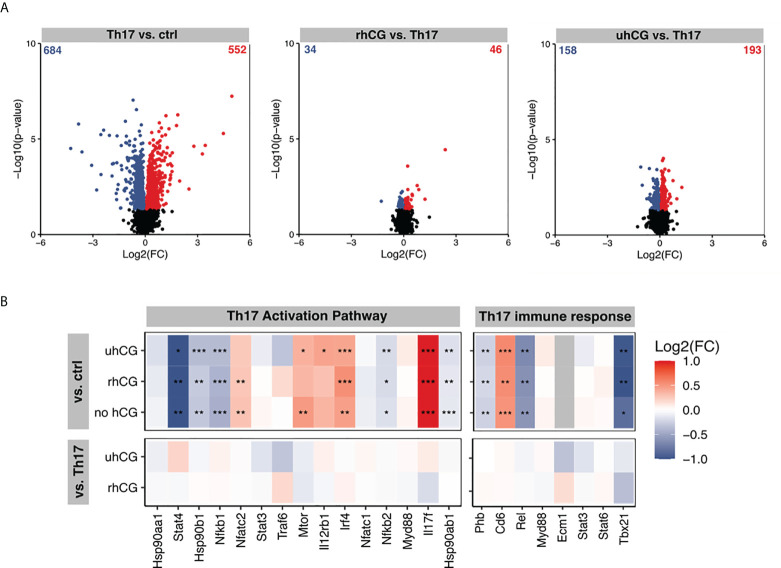
rhCG and uhCG did not alter molecules of the Th17 activation pathway and Th17 immune response. Naïve CD4^+^ T cells were polarized into Th17 cells for 3 days in the presence of 250 IU/ml uhCG or 500 mIU/ml rhCG. Differentiated cells cultured in the absence of hCG or non-polarized cells served as controls. Afterwards, proteome analyses were performed. **(A, B)** Overview of regulated proteins in Th17-polarized cells. **(A)** Summary of obtained Log2(FCs) and –Log10 (p-values) after naïve T cells were polarized into Th17 cells in the presence or absence of rhCG or uhCG. Significantly altered proteins were highlighted either blue (p 0.05, Log2 FC < 0) or red (p 0.05, Log2 FC > 0) (*n* = 4). **(B)** Ingenuity pathway analyses (IPA) of molecules involved in the Th17 activation pathway and immune response. * indicates *p <*0.05; ** indicates *p <*0.01; *** indicates *p <*0.001. rhCG, recombinant hCG; uhCG, urine-derived hCG.

Next, we investigated the effects of hCG on Th17 cell functionality and differentiation. To this end, isolated naïve T cells were polarized into Th17 cells under Th17-differentiating conditions for 3 days in the presence or absence of rhCG (50mIU/ml, 100mIU/ml, 500mIU/ml) or uhCG (100IU/ml, 250IU/ml, 500IU/ml). The cells were then analyzed by flow cytometry, with a gating strategy and representative dot plots of markers and FMO controls shown in **Figure 2A**. Both hCG preparations interfered with Th17 differentiation, resulting in significantly lower percentages of CD4^+^IL-17^+^ and CD4^+^RORγt^+^ cells among viable CD45^+^ T cells than in the absence of hCG (**Figures 2B, C, G, H**). This regulation seemed to be concentration-dependent, as more pronounced effects were observed at higher hCG concentrations. Neither rhCG nor uhCG significantly altered the frequency of CD4^+^FOXP3^+^ Treg cells under Th17-promoting conditions (**Figures 2D, I**), but both hCG preparations resulted in significantly lower percentages of CD4^+^TNFα^+^ T cells than in the absence of hCG (**Figures 2E, J**). Although the frequencies of CD4^+^IL-10^+^ T cells seemed to increase dose-dependently with rhCG or uhCG treatment, these apparent effects were not statistically significant (**Figures 2F, K**). To further uncover potential intracellular molecules that are regulated by hCG and may explain reduced Th17 differentiation, we performed proteomic analyses in Th17-polarized and non-polarized cells in the presence or absence of rhCG or uhCG. In total, 3897 proteins were reliably identified, of which 552 proteins were up-regulated and 684 proteins were down-regulated in Th17-polarized cells compared to non-polarized cells. Presence of rhCG and uhCG provoked an up-regulation of 46 and 193 proteins and a down-regulation of 34 and 158 proteins, respectively, compared to non-hCG-treated cells (**Figure 3A**). By particularly focusing on molecules involved in the Th17 differentiation pathway and immune response, in Th17-polarized cells, we found molecules promoting Th17 generation and functionality such as Irf4 (45), IL-12rb1 (46), Mtor (47), Cd6 (48) and IL-17f (49) to be up-regulated and molecules hindering Th17 activity such as Stat4 (50), Nfkb1 (51) and Tbx21 (52) to be down-regulated. However, neither rhCG nor uhCG did significantly alter the abundance of those molecules (**Figure 3B**). Based on these data we propose that hCG efficiently hampers Th17 cell generation. However, molecules participating in this process could not be identified by our current analyses.

### IL-2 blockage abrogates hCG-mediated interference with Th17 differentiation

**Figure 4:**
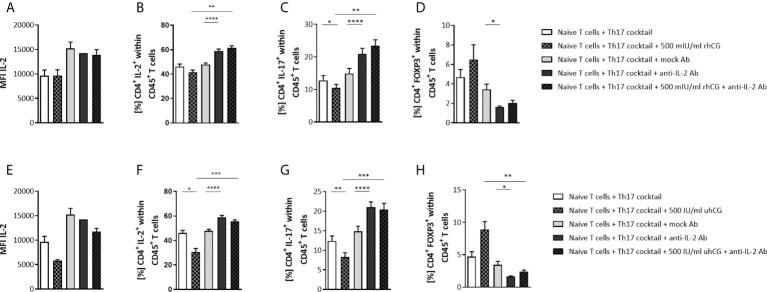
IL-2 blockage prevented the inhibition of Th17 differentiation by hCG *in vitro.* Naïve CD4^+^ T cells were isolated by magnetic-activated cell sorting (MACS) and polarized into Th17 cells under Th17-differentiating conditions for 3 days *in vitro*, in the presence or absence of 500 IU/ml uhCG or 500 mIU/ml rhCG, and anti-mouse IL-2 mAb or IgG2a isotype control mAb (mock Ab), respectively. Percentages of different T cell subsets as well as intracellular cytokine production were determined by flow cytometry. **(A, E)** Graphs showing intracellular IL-2 production in CD4^+^ T cells expressed as the mean MFI. **(B–D, F–H)** Graphs showing percentages of CD4^+^IL-2^+^, CD4^+^IL-17^+^ and CD4^+^FOXP3^+^ cells within CD45^+^ cells. Naïve T cells from WT (*n* = 6) female mice were included in the assays. All assays were run in duplicate. Data are presented as the mean plus standard error of the mean (S.E.M.). Statistical analysis among groups was performed using repeated measures one-way ANOVA followed by Sidak’s multiple-comparison test. Following, means plus S.E.M. are provided for significant differences between groups for CD4^+^IL-2^+^: 41.44 ± 1.921 vs 61.44 ± 1.799 (500mIU rhCG vs 500mIU rhCG plus anti-IL-2), 47.87 ± 1.251 vs 58.94 ± 1.567 (mock Ab vs anti-IL-2); 46.13 ± 2.104 vs 30.42 ± 3.153 (w/o hCG vs 500IU uhCG), 30.42 ± 3.153 vs 55.58 ± 1.246 (500IU uhCG vs 500IU uhCG plus anti-IL-2), 47.87 ± 1.251 vs 58.91 ± 1.567 (mock Ab vs anti-IL-2); for CD4^+^IL-17^+^: 12.81 ± 1.461 vs 10.46 ± 1.085 (w/o hCG vs 500mIU rhCG), 10.46 ± 1.085 vs 23.34 ± 1.892 (500mIU rhCG vs 500mIU rhCG plus anti-IL-2), 14.87 ± 1.574 vs 20.90 ± 1.706 (mock Ab vs anti-IL-2), 12.41 ± 1.257 vs 8.271 ± 1.101 (w/o hCG vs 500IU uhCG), 8.271 ± 1.101 vs 20.38 ± 1.609 (500IU uhCG vs 500IU uhCG plus anti-IL-2), 14.87 ± 1.285 vs 21.00 ± 1.396 (mock Ab vs anti-IL-2); for CD4^+^FOXP3^+^: 3.434 ± 0.5329 vs 1.628 ± 0.09477 (mock Ab vs anti-IL-2), 8.879 ± 1.225 vs 2.403 ± 0.2150 (500IU uhCG vs 500IU uhCG plus anti-IL-2), 3.434 ± 0.5329 vs 1.628 ± 0.09477 (mock Ab vs anti-IL-2). * indicates *p <*0.05; ** indicates *p <*0.01; *** indicates *p <*0.001; **** indicates *p <*0.0001. rhCG, recombinant hCG; uhCG, urine-derived hCG.

hCG is known to stimulate IL-2 production (53), and IL-2 can block Th17 differentiation and induce generation of Treg cells (54), suggesting that hCG may participate in the maintenance of the Th17–Treg balance *via* IL-2. The roles of IL-2 and hCG were therefore investigated by stimulation of Th17 differentiation for 3 days in the presence of an IL-2 blocking Ab and either 500 IU/ml uhCG or 500 mIU/ml rhCG followed by flow cytometry analyses. MFI measurements demonstrated that the hCG treatments and IL-2 blockage did not significantly affect intracellular IL-2 expression (**Figures 4A, E**), and cytometric bead array (CBArray) analyses of cell-culture supernatants showed that hCG treatments did not affect IL-2 secretion (data not shown). However, uhCG, but not rhCG, was able to significantly reduce the frequencies of CD4^+^IL-2^+^ T cells (**Figures 4B, F**). IL-2 blockage significantly augmented CD4^+^IL-2^+^ T cell frequencies when compared to mock Ab treatment and the combined treatment of hCG and anti-IL-2 extinguished the hCG-mediated effect (**Figures 4B, F**). The inhibitory effect of both hCG preparations on Th17 polarization was confirmed (**Figures 4C, G**). IL-2 blockage resulted in significantly higher IL-17 frequencies than treatment with a mock Ab control, and the combinations of hCG and IL-2 blockage abrogated the inhibitory effects of hCG on Th17 differentiation (**Figures 4C, G**). By contrast, blockage of IL-2 prevented Treg cell differentiation (**Figures 4D, H**). The reduced potential of naïve T cells to differentiate into Treg cells when IL-2 was missing was not reversed by the addition of hCG (**Figures 4D, H**). Aiming to provide a deeper insight into the mechanism of hCG-mediated Th17 differentiation impairment, our results suggest that IL-2 blockage can interfere with hCG effect on Th17 differentiation, although hCG itself seems not to affect intra- and extracellular IL-2 expression.

### rhCG and uhCG transdifferentiate Th17 cells into Th subsets with an anti-inflammatory profile

**Figure 5:**
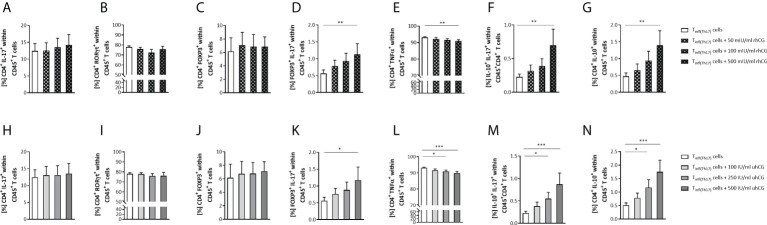
rhCG and uhCG induced differentiation of Th17 cells to anti-inflammatory subtypes. Naïve CD4^+^ T cells were isolated by magnetic-activated cell sorting (MACS) and polarized into Th17 cells under Th17-differentiating conditions for 3 days *in vitro*. Following they were exposed to uhCG (100 IU/ml, 250 IU/ml or 500 IU/ml) or rhCG (50 mIU/ml, 100 mIU/ml or 500 mIU/ml) for 24 h under continuing Th17-differentiating conditions. Percentages of different T cell subsets as well as intracellular cytokine production were determined by flow cytometry. **(A–N)** Graphs showing the percentages of CD4^+^IL-17^+^, CD4^+^RORγt^+^, CD4^+^FOXP3^+^, FOXP3^+^IL-17^+^, CD4^+^TNFα^+^, IL-10^+^IL-17^+^ and CD4^+^IL-10^+^ cells within CD45^+^ T cells in the rhCG **(A–G)** or uhCG **(H–N)** treatment groups. T_inf(Th17)_ cells from WT (*n* = 5) female mice were included in the assays. All assays were run in duplicate. Data are presented as the mean plus standard error of the mean (S.E.M.). Statistical analysis among groups was performed using the Friedman test followed by Dunn’s multiple-comparison test. Following, means plus S.E.M. are provided for significant differences between groups for FOXP3^+^IL-17^+^: 0.5617 ± 0.1064 vs 1.135 ± 0.3112 (w/o hCG vs 500mIU rhCG), 0.5609 ± 0.1068 vs 1.173 ± 0.3895 (w/o hCG vs 500IU uhCG); for CD4^+^TNFα^+^: 93.11 ± 0.4625 vs 91.00 ± 0.7252 (w/o hCG vs 500mIU rhCG), 93.11 ± 0.4625 vs 90.94 ± 0.7986 (w/o hCG vs 250IU uhCG), 93.11 ± 0.4625 vs 89.87 ± 1.019 (w/o hCG vs 500IU uhCG); for IL-10^+^IL-17^+^: 0.2278 ± 0.04261 vs 0.6995 ± 0.2387 (w/o hCG vs 500mIU rhCG), 0.2278 ± 0.04261 vs 0.5502 ± 0.1425 (w/o hCG vs 250IU uhCG), 0.2278 ± 0.04261 vs 0.8713 ± 0.2508 (w/o hCG vs 500IU uhCG); for CD4^+^IL-10^+^: 0.4719 ± 0.1017 vs 1.394 ± 0.4336 (w/o hCG vs 500mIU rhCG), 0.5119 ± 0.08716 vs 1.168 ± 0.2930 (w/o hCG vs 250IU uhCG), 0.5119 ± 0.087116 vs 1.748 ± 0.4320 (w/o hCG vs 500IU uhCG). * indicates *p <*0.05; ** indicates *p <*0.01; *** indicates *p <*0.001. rhCG, recombinant hCG; uhCG, urine-derived hCG.

In addition to interfering with the generation of Th17 cells, hCG may also cause phenotypic modulation of Th17 cells after full differentiation. To investigate the effects of hCG on Th17 plasticity, naïve T cells were treated to polarize to Th17 cells for 3 days and then exposed to rhCG (50mIU/ml, 100mIU/ml, 500mIU/ml) or uhCG (100IU/ml, 250IU/ml, 500IU/ml) for 24 h under continuing Th17-differentiating conditions. Neither rhCG nor uhCG altered the frequencies of Th17 cells (CD4^+^IL-17^+^ or CD4^+^RORγt^+^) (**Figures 5A, H, B, I**), or the frequencies of Treg cells (**Figures 5C, J**). However, treatment with either rhCG or uhCG resulted in significant, dose-dependent increases in the frequencies of FOXP3^+^IL-17^+^ cells (**Figures 5D, K**). Significant reductions in the frequencies of CD4^+^TNFα^+^ cells occurred in cultures exposed to high hCG concentrations (**Figures 5E, L**). Moreover, significant, dose-dependent increases in the frequencies of IL-10^+^IL-17^+^ and CD4^+^IL-10^+^ cells occurred in response to treatment with rhCG or uhCG (**Figures 5F, M, G, N**). In addition to the inhibitory effect of hCG on Th17 cell differentiation, these findings propose hCG to shift proinflammatory Th17 cells into Th cells with an anti-inflammatory phenotype.

### Adoptive transfer of proinflammatory T cells, including high levels of RORγt^+^ Th17 cells, into healthy pregnant female mice provokes fetal rejection, which is mitigated by prior hCG treatment

**Figure 6:**
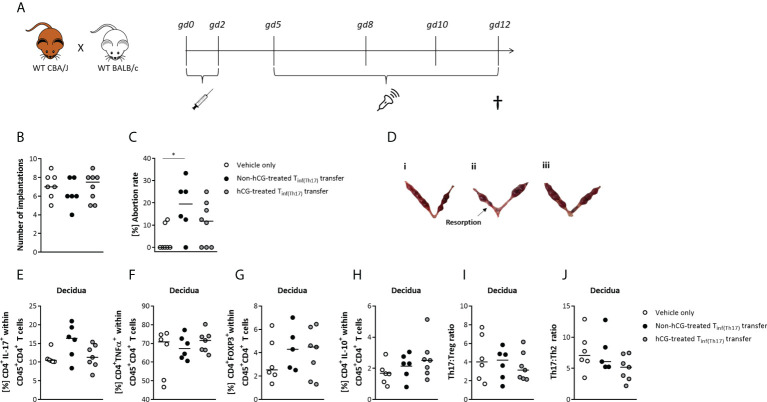
Adoptive transfer of T_inf(Th17)_ cells induced fetal rejection, but was partially prevented by hCG treatment during Th17 differentiation. Naïve CD4^+^ T cells were isolated by magnetic-activated cell sorting (MACS), polarized into Th17 cells under Th17-differentiating conditions for 3 days *in vitro*, in the presence or absence 500 mIU/ml rhCG, and then injected into pregnant CBA/J females during the first 2 days of gestation. **(A)** Overview of experimental setup, including mating of WT CBA/J females with WT BALB/c males, adoptive transfer of T_inf(Th17)_ cells (gd 0–2), high-frequency ultrasonography examination (gd 5, 8, 10, 12), euthanasia and tissue processing (gd 12). Experimental groups were vehicle-only injection (*n* = 7), non-hCG-treated T_inf(Th17)_ transfer (*n* = 6), and hCG-treated T_inf(Th17)_ transfer (*n* = 8), but for T-cell evaluation, *n* = 6, 6 and 7, respectively. **(B)** Total numbers of implantations. **(C)** Abortion rates. **(D)** Examples of excised bicornuate uteri from vehicle-only (i), non-hCG-treated (ii) and hCG-treated (iii) groups. **(E–J)** Percentages of CD4^+^IL-17^+^, CD4^+^TNFα^+^, CD4^+^FOXP3^+^ and CD4^+^IL-10^+^ cells within CD45^+^ T cells, as well as the Th17:Treg ratio (CD4^+^IL-17^+^:CD4^+^FOXP3^+^) and Th17:Th2 ratio (CD4^+^IL-17^+^:CD4^+^IL-10^+^) within decidual tissue on gd 12. Data are presented as medians showing individual values for each animal. For comparisons between experimental groups, the Kruskal–Wallis test followed by Dunn’s multiple-comparison test was applied. Following, medians are provided for significant differences between groups for Abortion rates: 0.00 vs 19.50 (vehicle only vs non-hCG-treated Th17 cells). * indicates *p <*0.05. gd, gestational day.

After we provided evidence for an *in vitro* modulation of Th17 cells by hCG, we next aimed to investigate a) potential harmful effects of Th17 cells on fetal survival and development *in vivo*, and sought to b) understand whether prior hCG treatment *in vitro* may alter Th17 action. Th17 cells were adoptively transferred into healthy pregnant female mice at very early pregnancy stages, following the time-course shown in **Figure 6A**. The proinflammatory Th17 cells that were transferred into pregnant female mice were derived from naïve T cells polarized into Th17 cells for 3 days with or without simultaneous treatment with 500 mIU/ml rhCG. These proinflammatory T cells are referred to herein as T_inf(Th17)_ cells, and included up to 60% RORγt^+^ Th17 cells, as well as >80% TNFα^+^ cells. Following transfer of T_inf(Th17)_ cells during the first two days of gestation, the number of implantations did not significantly alter compared with that in mice treated with vehicle only (PBS), although a trend of reduction in the number of implantations following transfer of T_inf(Th17)_ cells was noticeable (**Figure 6B**). An increased number of mice might strengthen that trend to statistical significance. Notably, transfer of T_inf(Th17)_ cells (but not of rhCG-treated T_inf(Th17)_ cells) significantly impaired fetal survival compared with vehicle-only controls (**Figures 6C, D**). Flow-cytometric analyses of different Th-cell subsets in decidual tissue samples taken from the fetal–maternal interface revealed no significant differences in the frequencies of proinflammatory CD4^+^IL-17^+^ or CD4^+^TNFα^+^ cells, or of anti-inflammatory CD4^+^FOXP3^+^ or CD4^+^IL-10^+^ cells between experimental groups (**Figures 6E–H**). However, the frequency of decidual CD4^+^IL-17^+^ cells was slighter higher, albeit not significant, following transfer of T_inf(Th17)_ cells than following transfer of hCG-treated T_inf(Th17)_ cells (**Figure 6E**). Similarly, frequencies of CD4^+^FOXP3^+^ cells following T_inf(Th17)_ cell transfer were not significantly different from those following vehicle-only treatment (**Figure 6G**). Th17:Treg and Th17:Th2 ratios in the decidua were not altered by T_inf(Th17)_ cell transfer (**Figures 6I, J**). Similarly, the frequencies and ratios of Th17, Treg, Th1 and Th2 cells in the spleen and draining lymph nodes were not significantly affected by T_inf(Th17)_ cell transfer (**Supplementary Figure 2**). Our data suggest that proinflammatory T cells, including high proportions of proinflammatory Th17 cells negatively affect fetal survival, which is not caused by significant changes in local and peripheral immune cell compartments. Previous hCG treatment is suggested to attenuate detrimental Th17 cell effects on fetal outcome.

### Adoptive T_inf(Th17)_ cell transfer impairs fetal growth regardless of the previous treatment with hCG

**Figure 7:**
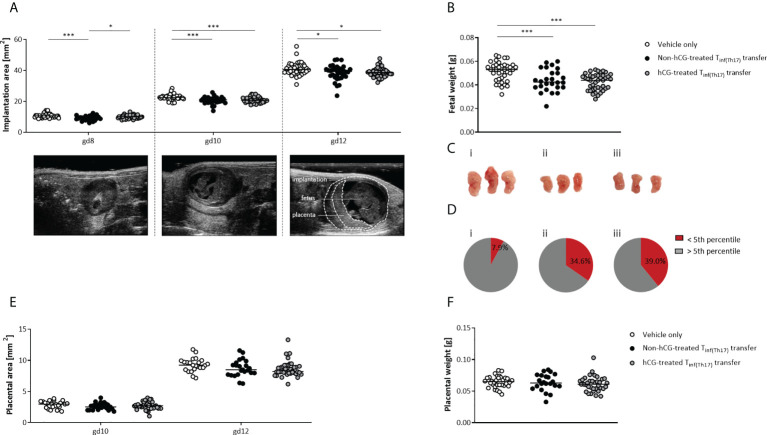
Adoptive transfer of T_inf(Th17)_ cells, with or without hCG treatment during Th17 differentiation, impaired fetal growth. Naïve CD4^+^ T cells were isolated by magnetic-activated cell sorting (MACS), polarized into Th17 cells under Th17-differentiating conditions for 3 days *in vitro*, in the presence or absence 500 mIU/ml rhCG, and then injected into pregnant CBA/J females during the first 2 days of gestation. **(A)** Implantation areas on gd 8, 10 and 12, determined by high-frequency ultrasonography. Examples of ultrasonography of individual implantations on each day are also shown. **(B)** Fetal weights on gd 12. **(C)** Examples of fetuses from vehicle-only (*n* = 7) (i), non-hCG-treated (*n* = 6) (ii) and hCG-treated (*n* = 8) (iii) groups on gd 12. **(D)** Percentages of fetuses with weights below the fifth percentile (intrauterine growth restriction) or above the fifth percentile on gd 12. **(E)** Placental areas on gd 10 and 12 measured by ultrasonography. **(F)** Placental weights on gd 12. Data are presented as medians showing individual values for each implantation/fetus/placenta. For comparisons between experimental groups, a mixed linear model using the final test principle was applied. Following, medians are provided for significant differences between groups for Implantation areas: 10.64 vs 8.942 (gd8), 22.61 vs 21.00 (gd10), 40.67 vs 39.18 (gd12) (vehicle only vs non-hCG-treated Th17 cells), 22.61 vs 20.71 (gd10), 40.67 vs 38.64 (gd12), (vehicle only vs hCG-treated Th17 cells), 8.942 vs 10.10 (gd8) (non-hCG-treated Th17 vs hCG-treated Th17 cells); for Fetal weights: 0.053 vs 0.0425 (vehicle only vs non-hCG-treated Th17 cells), 0.053 vs 0.044 (vehicle only vs hCG-treated Th17 cells). * indicates *p <*0.05; *** indicates *p <*0.001. gd, gestational day.

Next, we sought to dissect whether the deleterious effects of T_inf(Th17)_ cell transfer on fetal well-being also included impairment in fetal growth. Serial ultrasonographic measurements demonstrated significantly smaller implantation sizes on gestational days (gds) 8, 10 and 12 after the transfer of non-hCG-treated T_inf(Th17)_ cells compared with vehicle-only controls (**Figure 7A**). With transfer of hCG-treated T_inf(Th17)_ cells, the implantation size was not affected at gd 8, but was significantly lower at gd 10 and 12 than with vehicle-only controls (**Figure 7A**). Fetal weights were also significantly lower (**Figure 7B**) and fetuses smaller (**Figure 7C**) at gd 12 following transfer of hCG-treated and non-hCG-treated T_inf(Th17)_ cells than in vehicle-only controls. Severe growth restriction (defined by growth below the fifth percentile) occurred in 34.6% and 39.0% of fetuses following transfer of non-hCG-treated and hCG-treated T_inf(Th17)_ cells, respectively, compared with 7.9% of fetuses in the vehicle-only group (**Figure 7D**). The placental area (**Figure 7E**), thickness and diameter (data not shown) on gd 10 and 12, and placental weight on gd 12 (**Figure 7F**) were not affected by T_inf(Th17)_ cell transfer. These findings imply that proinflammatory T cells, including high levels of proinflammatory Th17 cells, compromise fetal growth in early-to-mid-pregnancy stages resulting in severe growth restriction later on.

### Impaired fetal growth following adoptive Th17 cell transfer is associated with abnormal maternal uterine-artery Doppler velocities

**Figure 8:**
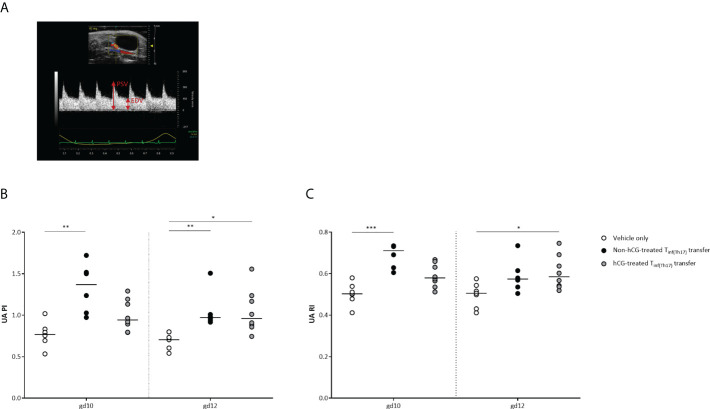
Adoptive transfer of T_inf(Th17)_ cells resulted in abnormal blood-flow parameters in the maternal UA. Naïve CD4^+^ T cells were isolated by magnetic-activated cell sorting (MACS), polarized into Th17 cells under Th17-differentiating conditions for 3 days *in vitro*, in the presence or absence 500 mIU/ml rhCG, and then injected into pregnant CBA/J females during the first 2 days of gestation. **(A)** Example of a Doppler flow diagram of the maternal UA, visualizing peak systolic velocity (PSV) and end-diastolic velocity (EDV). **(B)** Pulsatility index (PI) of the UA on gestational days (gd) 10 and 12 shown for vehicle-only (*n* = 7), non-hCG-treated (*n* = 6) and hCG-treated (*n* = 8) groups. **(C)** Resistance index (RI) of the UA on gd 10 and 12. Data are presented as medians, showing individual values for each animal. For comparisons between experimental groups, the Kruskal–Wallis test followed by Dunn’s multiple-comparison test was used. Following, medians are provided for significant differences between groups for UA PI: 0.7677 vs 1.369 (gd10), 0.7046 vs 0.9715 (gd12) (vehicle only vs non-hCG-treated Th17 cells), 0.7046 vs 0.9601 (gd12) (vehicle only vs hCG-treated Th17 cells); for UA RI: 0.5027 vs 0.7107 (gd10) (vehicle only vs non-hCG-treated Th17 cells), 0.5055 vs 0.5847 (gd12) (vehicle only vs hCG-treated Th17 cells). * indicates *p <*0.05; ** indicates *p <*0.01; *** indicates *p <*0.001. UA, uterine artery.

Following our previous observations, we next intended to study whether IUGR after adoptive T_inf(Th17)_ cell transfer is associated with pathological changes in the uteroplacental circulation (55). Uterine and umbilical Doppler sonography enables analysis of uteroplacental and fetoplacental circulations, facilitating identification of conditions associated with adverse pregnancy outcomes, such as IUGR, which is indicated by high vascular impedance, as quantified by high values for the pulsatility index (PI) and resistance index (RI) (56, 57). We performed Doppler measurements in the maternal uterine artery (UA) on gd 10 and 12 (**Figure 8A**). Measurements of the umbilical arteries (UmA) were not performed because of the absence of end-diastolic velocity (EDV) up to the second half of the murine pregnancy. Notably, both PI and RI were significantly higher in the UA on gd 10 and 12 following transfer of non-hCG-treated T_inf(Th17)_ cells than in vehicle-only controls. Transfer of hCG-treated T_inf(Th17)_ cells also resulted in significantly higher PI and RI on gd 12, compared with the controls (**Figures 8B, C**). Our results indicate an association between IUGR and pathological conditions in the uteroplacental circulation after transfer of proinflammatory T cells comprising high proportions of proinflammatory Th17 cells.

### Adoptive T_inf(Th17)_ cell transfer is associated with inadequate vascular remodeling of the uSAs

**Figure 9:**
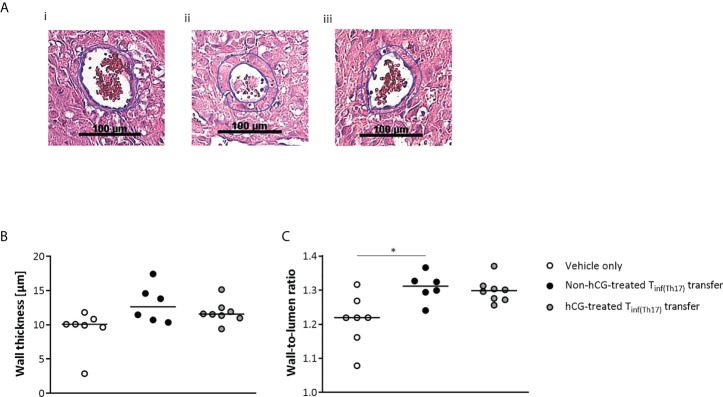
Adoptive transfer of T_inf(Th17)_ cells impaired vascular remodeling in the uSAs. Naïve CD4^+^ T cells were isolated by magnetic-activated cell sorting (MACS), polarized into Th17 cells under Th17-differentiating conditions for 3 days *in vitro*, in the presence or absence 500 mIU/ml rhCG, and then injected into pregnant CBA/J females during the first 2 days of gestation. **(A)** Examples of hematoxylin–eosin staining of sections through individual uSAs in the decidua basalis of animals in the vehicle-only (*n* = 7) (i), non-hCG-treated (*n* = 6) (ii) and hCG-treated (*n* = 8) (iii) groups on gd 12, viewed at a magnification of 200×. **(B)** Medial uSA wall thickness, calculated from 3–8 uSAs per animal. **(C)** Medial uSA wall-to-lumen ratio, calculated from 3–8 uSAs per animal. Data are presented as medians, with individual values for each animal. For comparisons between experimental groups, the Kruskal–Wallis test followed by Dunn’s multiple-comparison test was used. Following, medians are provided for significant differences between groups for the wall-to-lumen ratio: 1.219 vs 1.312 (vehicle only vs non-hCG-treated Th17 cells). * indicates *p <*0.05. uSA, uterine spiral artery.

Inadequate remodeling of uSAs has been shown to result in high resistance vessels (58), which could account for the elevation of vascular indices that we observed in the maternal UA. We therefore assessed uSA remodeling after adoptive T_inf(Th17)_ cell transfer, as indicated in **Figure 9A**. Measurement of uSA-wall thickness revealed no significant differences among the groups (**Figure 9B**). However, the wall-to-lumen ratios were higher after adoptive T_inf(Th17)_ cell transfer than in the controls, and this difference was significant with the transfer of non-hCG-treated T_inf(Th17)_ cells (**Figure 9C**). These data propose that transfer of proinflammatory T cells, and in particular proinflammatory Th17 cells result in an insufficiency of uSA remodeling, leading to high resistance vessels, undernourishment of the fetus, and in consequence IUGR.

## Discussion

Normal pregnancy represents a physiological state of immune tolerance in which two genetically nonidentical individuals co-exist without inducing immunological rejection. It is now well documented that already at early pregnancy stages, various tolerance mechanisms are activated, with evidence that the fetus itself actively secretes factors to support its acceptance and growth in the maternal womb. One of these factors is the pregnancy hormone hCG (59–61).

Here, we aimed to study how the placental hormone hCG influences Treg and Th17 cells and their interplay during pregnancy. Therefore, we analyzed the effects of uhCG and rhCG on Treg cells, on Th17 generation and on Th17 plasticity *in vitro*. To have a full picture of the triangle Treg/Th17/hCG, we complemented our already published studies on hCG-treatment of T and Treg cells (59, 60, 62) with *in vivo* studies addressing hCG-mediated Th17 effects in a murine pregnancy model *in vivo*. We conclude that hCG promotes the generation of murine pregnancy-protective Treg cells and hinders the differentiation of proinflammatory Th17 cells that are detrimental for fetal well-being. This is supported by our *in vitro* findings that hCG enhances Treg proportions, inhibits Th17 differentiation, and forces the transformation of proinflammatory Th17 cells into Th17 subsets with anti-inflammatory phenotypes. *In vivo*, transferred proinflammatory Th17 cells into healthy pregnant females promoted fetal rejection, impaired fetal growth and sufficient remodeling of uSAs, and induced abnormal blood flow velocities. Transfer of Th17 cells that were treated with hCG during their differentiation culminated in milder effects and partial alleviated the harmful *in vivo* effect of proinflammatory Th17 cells on pregnancy. This suggest a strong potential of hCG in reprogramming inflammatory cells for their use in therapeutic approaches for pregnancy pathologies.

Results from our previous studies in humans and mice suggested that hCG supports Treg cell enrichment at the fetomaternal interface by recruitment of these cells from the periphery and by induction of Treg generation from naïve Th cells (59, 62). Further results suggested that hCG increases the suppressive activity of Treg cells and promotes the secretion of immunosuppressive cytokines (60). In the current study, we found that treatment of CD4^+^CD25^+^ T cells with uhCG, but not with rhCG, increased the proportions of FOXP3^+^ Treg cells expressing CD25, CTLA-4, PD-1 and ICOS. Notably, expression levels of these markers in individual cells did not change in response to hCG. The purity of CD4^+^CD25^+^FOXP3^+^ Treg cells directly after isolation was >90%, but <100%. Thus, based on these findings (shown in **Figure 1**), we propose that uhCG promotes conversion of resting non-Treg cells into Treg cells, or expansion of pre-existing Treg cells, rather than increasing cellular expression levels of the markers. These markers are all important for Treg cell function during pregnancy, enabling these cells to limit the harmful effects of alloantigen-specific effector T cells and other immune-cell populations at the fetomaternal interface, and to protect the fetus from rejection, as discussed elsewhere (63, 64). Secretion of IL-10 and TGF-β by Treg cells has similarly been shown to regulate effector T cells through induction of apoptosis and suppression of cytotoxicity (as reviewed previously (63)). We have now shown that hCG is also involved in this pathway, as rhCG stimulates TGF-β secretion. Differences in the composition of uhCG and rhCG presumably explain their differential effects on molecules associated with Treg cell suppressive activity and on several aspects of human and mouse Treg cell biology *in vitro* (62), although notably uhCG and rhCG promote similar patterns of Treg cell activation. Whether uhCG and rhCG vary in their capacities to regulate immune cells *in vivo* in patients undergoing IVF is still a matter for debate and needs further investigation.

Human and mouse conventional T cells, as well as Treg cells, express the LH/CGR (59, 60, 65–67), suggesting a pathway for regulation by hCG. Diao and colleagues proposed that hCG induces differentiation of human naïve T cells into Treg cells though the LH/CGR (67). We confirmed that hCG acts through the conventional receptor by employing LH/CGR mice. In agreement with Diao´s theory, we found that uhCG did not increase the proportions of Treg cells in the absence of the LH/CGR. Moreover, compared with WT cells, we observed reduced proportions of Treg cells expressing CTLA-4 or PD-1, and diminished expression of CTLA-4, PD-1 and ICOS in cells lacking LH/CGR. The isolation of Treg cells from WT and LH/CGR-deficient female mice resulted in similar yields of CD4^+^CD25^+^FOXP3^+^ Th cells (data not shown), suggesting that differences between these mice result from impairment of the suppressive functions of Treg cells in LH/CGR-deficient mice rather than from reduction of Treg cell numbers. Functional assays with isolated Treg cells from both genotypes of mice are now required to determine the validity of this assumption.

Our results demonstrated dose-dependent impairment of murine Th17 differentiation by both uhCG and rhCG. However, the precise intracellular pathways underlying this effect remain to be elucidated. Previously, hCG has been also shown to efficiently block human Th17 differentiation *in vitro* (68). Additionally, we found that both hCG preparations prevented elevation of the frequency of TNFα^+^ Th cells, and favored differentiation into IL-10^+^ Th cells, under Th17-promoting conditions. Results from several experiments indicated that IL-2 blockage interfered with hCG-mediated inhibition of Th17 differentiation, although, in contrast to previous observations (53), we did not find any hCG-stimulated increase in IL-2 production. Notably, IL-2 blockage impeded Treg cell generation, which may contribute to the generation of more Th17 cells (69).

In addition to the effects on Th17 differentiation, we observed effects of both hCG preparations on Th17 plasticity. We suggest that hCG, instead of completely transdifferentiating Th17 cells into another Th subtype, induces regulatory (FOXP3) and anti-inflammatory (IL-10) properties within the Th17 population, promoting immunosuppressive phenotypes that can function in the resolution of immune responses or even participate in the induction of immune tolerance. In both humans and mice, Th cells co-expressing IL-17 and FOXP3 have been shown to possess immunosuppressive activities *in vitro* (70, 71). In the same way, human Th cells co-expressing IL-17 and IL-10 can act as modulators, expressing a variety of immunoregulatory molecules and polarizing monocytes into M2 macrophages (72). In our experiments, no significant changes occurred in the total numbers of IL-17^+^ and RORγt^+^ Th17 cells or FOXP3^+^ Treg cells after hCG exposure, indicating that hCG did not provoke a complete conversion of Th17 cells into FOXP3^+^ Treg cells. Notably, no evidence exists for such a complete conversion (61). However, we found that hCG exposure significantly increased the frequencies of cells co-expressing IL-17 with IL-10, as well as IL-17 with FOXP3, suggesting the induction of anti-inflammatory, immunotolerant Th17 phenotypes. Previously, Th17 cells have been shown to transdifferentiate into IL-10^+^FOXP3^−^ Tr1 cells under self-limiting inflammatory conditions (73). We found that exposure of Th17 cells to hCG resulted in a significant elevation of the frequency of CD4^+^IL-10^+^ Th cells in a population in which >90% of the cells were FOXP3^−^, which could indicate induction of Tr1 or Th2 cells. However, as the total Th17 numbers did not decline, hCG does not seem to have driven a conversion of proinflammatory Th17 cells into Tr1 and/or Th2 cells. Collectively, these results suggest that hCG not only prevents the *de novo* generation of proinflammatory Th17 cells, but also forces the trans-differentiation of pre-existing proinflammatory Th17 cells into Th17 cells with a more anti-inflammatory phenotype.

On the basis of these results, we speculated that hCG treatment of Th17 cells might dampen their potentially destructive activity during pregnancy. To test our Th17 hypothesis, we adoptively transferred proinflammatory Th cells (produced with or without hCG during Th17 differentiation) into healthy pregnant females, and studied the pregnancy outcomes and fetal growth *in vivo*. The transferred cells produced without hCG contained up to 60% CD4^+^RORγt^+^ Th17 cells and <15% CD4^+^IL-17^+^ cells, as well as >80% TNFα-producing Th cells, resembling a pool of T cells with a strong proinflammatory phenotype and a majority of Th17 cells. By comparison, the transferred cells produced with hCG contained approximately half the frequencies of RORγt^+^ Th17 cells and IL-17-producing cells, as well as lower frequencies of TNFα-producing Th cells. The mouse model used prevented re-sorting of Th17 cells from cell cultures after hCG treatment and, accordingly, the transfer of pure Th17 cells into pregnant females. Hence, our *in vivo* data do not represent effects exclusively provoked by Th17 cells. However, we believe that our experimental setup convincingly mimics the physiological situation in which a strong inflammatory immune response, involving different proinflammatory Th subsets, counteracts the maintenance of fetal tolerance and results in impairment of fetal well-being. Results from multiple studies have shown that high-grade inflammation in pregnancy leads to severe pathologies, such as spontaneous abortion or pre-eclampsia (as summarized in (74) and (75)). In keeping with these previous observations, our data showed that adoptive transfer of non-hCG-treated T_inf(Th17)_ cells at early pregnancy stages resulted in fetal rejection. Previously, adoptive transfer of 1 × 10^6^ *in vitro*-generated Th17 cells (equal number of transferred cells as in our study) on gd 7.5 in a syngeneic mating combination also provoked significant fetal loss (76). Unfortunately, in that study the percentage of Th17 cells within the transferred T-cell pool was not assessed, so it is not possible to determine the contribution of Th17 cells to this effect. In addition, it should be noted that adaptive immune responses are relevant in an allogeneic rather than a syngeneic context. In the previous study, Th17 cells were also transferred *via* the tail vein, and they reached the decidual tissue and could still be detected 7 days after transfer (76). In our study, hCG treatment during Th17 differentiation attenuated the negative effects of the differentiated cells, although not completely preventing them. rhCG treatment halved the frequency of Th17 cells within the transferred T cell pool. However, we still transferred ~3 × 10^5^ RORγt^+^ and 5 × 10^4^ IL-17^+^ Th17 cells, seeming to be sufficient to affect fetal survival. Our results suggest that fetal rejection is not primarily caused by substantial immunological changes in the T cell compartments in peripheral lymphoid organs or directly at the fetal-maternal interface, as we did not observe significant alterations in Th subsets at these locations in animals with T_inf(Th17)_ cell transfer, compared with vehicle-only controls.

Our data indicate that pathological levels of proinflammatory Th cells and particularly Th17 cells negatively affect placentation, resulting in an undernourishment of the fetuses and consequently leading to IUGR. Although adoptive T_inf(Th17)_ cell transfer did not reduce placental size or weight, we observed a clear effect on uSA remodeling. Independent of exposure to rhCG during differentiation, T_inf(Th17)_ cells inhibited the adequate remodeling of uSAs that is a prerequisite to generation of low-resistance vessels that enable adequate placental blood flow. We also found higher PI and RI values in the UA following T_inf(Th17)_ cell transfer than in vehicle-only controls, indicating the presence of high-resistance vessels, with inadequate blood flow from the mother to the fetus. Presumably as a consequence of impairment of the blood supply, fetuses from pregnant females infused with T_inf(Th17)_ cells were small for gestational age, with >30% of them being severely growth restricted (below the fifth percentile). On the basis of findings derived from rat models, a pathophysiological role has been proposed for Th17 cells and IL-17 in pre-eclampsia and IUGR. Th17 cells, likely through IL-17 secretion, are thought to induce placental and renal oxidative stress, leading to the production of specific autoantibodies and placental vascular dysfunction (77). However, whether human proinflammatory Th17 cells induce the same pathological pathways in patients with pre-eclampsia is not yet known.

In conclusion, we suggest that hCG alters the anti-inflammtory:pro-inflammtory Treg : Th17 ratio in favor of anti-inflammation by reducing the number of newly generated Th17 cells as well as enhancing the plasticity of activated T cells to induce production of anti-inflammatory cytokines such as IL-10 and promoting the transformation of proinflammatory Th17 cells into Th17 cell subsets with anti-inflammatory profiles. *In vivo*, we confirmed the pathogenic role of proinflammatory Th17 cells on pregnancy outcomes, and provided new insights into the mechanisms underlying their harmful properties. *In vitro* inhibition of Th17 differentiation by hCG partly reduced the harmful effects of the transferred cells on fetal well-being. We believe that hCG treatment is a potential option for patients undergoing IVF or experiencing recurrent spontaneous abortion, and is a promising approach for improvement of pregnancy outcomes. This presumption is supported by a meta-analysis, which concluded that intrauterine hCG infusion in IVF patients before embryo transfer significantly improves implantation rates, clinical pregnancy rates and life birth rates, and significantly lowers the miscarriage rates (78). Remarkably, IVF patients who received hCG exhibited significantly elevated peripheral Treg cell levels when compared to non-hCG-treated controls (79) and spontaneous abortion patients showed lower peripheral numbers of Th17 cells and elevated Treg cell frequencies after hCG exposure (44, 80) underlining the clinical relevance of our current findings.

## Materials and methods

### Animals

WT CBA/J females and WT BALB/c males were purchased from Janvier Labs. LH/CGR-deficient CBA/J females were derived in-house from LH/CGR-deficient mice (mixed C57BL/6 and 129/SvEv background) provided as a gift by the group of Dr. Huhtaniemi (formerly University of Turku, Finland) (81). These mice were backcrossed for 10 generations on a CBA/J background to obtain LH/CGR-deficient CBA/J mice. All animals were maintained in our house-intern animal facility (Magdeburg, Germany) and kept under a 12 h light–12 h dark cycle at 22 ± 2°C, with air humidity of 40–60%. Water and food were provided *ad libitum*. *In vitro* analyses used 8-week-old virgin WT CBA/J and LH/CGR-deficient CBA/J females. Adoptive cell transfer was performed in 8-week-old pregnant WT CBA/J females that had been mated with 8–10-week-old WT BALB/c males. After mating, females were checked twice a day for the presence of a vaginal plug, which indicated gd 0.

### Ethics approval

All animal experiments were performed according to institutional guidelines with ministerial approval of the Landesverwaltungsamt Sachsen-Anhalt (AZ42502-2-1515 UniMD). Experiments were conducted by authorized personnel, according to the Guide for Care and Use of Animals in Agriculture Research and Teaching.

### Isolation and purity of naïve CD4^+^ T cells and CD4^+^CD25^+^ T cells

T cell subsets were isolated from a mixture of spleen and inguinal and para-aortic lymph nodes of virgin WT or LH/CGR-deficient CBA/J females (for CD4^+^CD25^+^ T cells) or WT-only (for CD4^+^ T cells). Naïve T cells and CD4^+^CD25^+^ T cells were isolated by magnetic-activated cell sorting (MACS) using the MojoSort Mouse CD4 Naïve T Cell Isolation Kit (BioLegend) or the CD4^+^CD25^+^ Regulatory T Cell Isolation Kit, mouse (Miltenyi Biotec). All steps were performed according to the manufacturers’ instructions under sterile conditions. The purity of isolated cell suspensions, as determined by flow cytometry, was >93% for naïve CD4^+^ T cells and >90% for CD4^+^CD25^+^ T cells.

### Th17 polarization of naïve CD4^+^ T cells

Naïve CD4^+^ T cells were forced to polarize into Th17 cells by 3 days of culture under Th17-differentiating conditions using a protocol and reagents provided by BioLegend (unless otherwise indicated). Briefly, isolated naïve CD4^+^ T cells were suspended in ‘full medium’, consisting of RPMI 1640 medium (ThermoFisher), supplemented with 10% FBS (Biochrom) and 1% penicillin/streptomycin (ThermoFisher), and added to multiwell plates precoated with anti-mouse CD3 mAb (clone 145-2C11, 5 µg/ml). To induce Th17 differentiation, recombinant IL-6 (50 ng/ml), recombinant human TGF-β (1 ng/ml), recombinant mouse IL-23 (5 ng/ml), anti-mouse IL-4 mAb (clone 11B11, 10 µg/ml), anti-mouse IFN-γ mAb (clone XMG1.2, 10 µg/ml) and anti-mouse CD28 mAb (clone 37.51, 5 µg/ml, BD Biosciences) were added to the cultures.

### hCG treatment of cultures of mouse CD4^+^CD25^+^ T cells, cells undergoing Th17 differentiation and differentiated Th17 cells

Isolated CD4^+^CD25^+^ T cells (5 × 10^4^ cells) were cultured in 250 µl full RPMI 1640 medium supplemented with recombinant mouse IL-2 (10 ng/ml, R&D Systems) and anti-mouse CD28 mAb (clone 37.51, 5 µg/ml) on multiwell plates precoated with anti-mouse CD3 mAb (clone 145-2C11, 3 µg/ml). Either 250 IU/ml uhCG (Sigma) or 100 mIU/ml rhCG (Merck Serono) was added, and the cultures incubated for 24 h. CD4^+^CD25^+^ T-cell cultures without addition of hCG served as controls. The concentration of uhCG was chosen according to physiological hCG levels found in women with healthy pregnancies during the first trimester (25–288 IU/ml during weeks 9–12, according to the American Pregnancy Association), and the rhCG concentration was chosen according to concentrations used for rhCG and other recombinant gonadotropins in previous studies (82, 83). Following culture, the frequencies of CD4^+^FOXP3^+^ Treg cells expressing PD-1, CTLA-4 or ICOS, as well as the MFI for each marker, were determined by flow cytometry. Additionally, the levels of IL-10, IL-35 and TGF-β in the culture supernatants were measured by ELISA.

Various concentrations of uhCG (100 IU/ml, 250 IU/ml and 500 IU/ml) or rhCG (50 mIU/ml, 100 mIU/ml and 500 mIU/ml) were added to samples of CD4^+^ T cells (2.5 × 10^5^ cells per sample) in 250 µl full RPMI 1640 medium under Th17-differentiating conditions for 3 days. Control cells were cultured in the absence of hCG. In addition, cells were cultured in Th17-differentiating conditions in the presence or absence of 500 IU/ml uhCG or 500 mIU/ml rhCG, with or without anti-mouse IL-2 mAb (clone JES6-1A12, 10 µg/ml, BioLegend) or IgG2a isotype control mAb (clone RTK2758, 10 µg/ml, BioLegend). Percentages of different T cell subsets, as well as MFI of IL-2, were determined by flow cytometry. To evaluate intracellular cytokine production by flow cytometry, T cells were stimulated with PMA (50 ng/ml, Sigma), ionomycin (500 ng/ml, ThermoFisher) and brefeldin A (10 µg/ml, BioLegend) for the final 6 h of culture. To evaluate IL-2 secretion in supernatants *via* CBArray, cells were stimulated with PMA and ionomycin in the absence of brefeldin A.

Naïve CD4^+^ T cells were polarized to Th17 cells under Th17-differentiating conditions for 3 days, then samples of 2.5 × 10^5^ differentiated cells (containing ~60% CD4^+^RORγt^+^ cells and ~15% IL-17-producing cells) in 250 µl full RPMI 1640 medium were exposed to uhCG (100 IU/ml, 250 IU/ml or 500 IU/ml) or rhCG (50 mIU/ml, 100 mIU/ml or 500 mIU/ml) for 24 h. During this period, cells were kept under Th17-differentiating conditions. Differentiated cells cultured in the absence of hCG served as controls. Cell plasticity was assessed by flow cytometry. To evaluate intracellular cytokine production, cells were stimulated with PMA (50 ng/ml), ionomycin (500 ng/ml) and brefeldin A (10 µg/ml) for the final 6 h of culture.

### Determination of cytokine secretion by ELISA or CBArray

Levels of IL-10, IL-35 and TGF-β were determined in supernatants from Treg cultures with the Quantikine ELISA Mouse IL-10 Immunoassay (R&D Systems), the LEGEND MAX Mouse IL-35 Heterodimer ELISA Kit or the LEGEND MAX Total TGF-β1 ELISA Kit (BioLegend). IL-2 levels in supernatants of differentiating Th17 cells were measured with the BD Cytometric Bead Array, Mouse Th1/Th2/Th17 Cytokine Kit (BD Biosciences). All steps were performed according to the manufacturers’ instructions.

### Sample preparation for LC-MS/MS-based proteomics

Naïve CD4^+^ T cells were polarized into Th17 cells for 3 days in the presence of 250 IU/ml uhCG or 500 mIU/ml rhCG. Differentiated cells cultured in the absence of hCG or non-polarized cells served as controls. After culture, cell pellets were washed trice with sterile PBS, dried and resuspended in 100 µl radioimmunoprecipitation assay buffer (RIPA buffer) containing 50 mM Tris-HCL (pH 7.4), 150 mM sodium chloride, 1% Triton-X100, 0.5% sodium deoxycholate and 0.1% sodium dodecyl sulfate to induce cell lysis. To prevent protein degradation and modification, a protease-inhibitor cocktail was added (cOmplete, Sigma). Cell lysates were kept on ice for 1 h, centrifuged (12.500 g, 10 min, 4°C) and supernatants were stored at -80°C. Protein concentration was determined by colorimetric assay (Bradford, ThermoFisher) at 595 nm.

For the proteome analyses an untargeted approach was applied using 30µg of protein lysate per sample, as described previously (84). Briefly, the lysate volume was adjusted to 110µl with 100mM triethylammonium bicarbonate (TEAB) (Sigma Aldrich, Germany), followed by protein reduction with 9.5mM tris-(2-carboxyethyl)-phosphin (TCEP) (Sigma Aldrich, Germany) for 1 h at 55°C. Protein Alkylation was performed with 17.05 mM iodoacetamide (IAA) (Merck, Germany) for 30 min at room temperature in the dark. Next, the samples were acidified using formic acid (Merck, Germany). After adding 120µl acetonitrile (ACN), proteins were loaded on 30µg SpeedBead™Magnetic Carboxylate modified particles (Sigma Aldrich, MO, USA). Samples were washed twice with 70% EtOH (Merck, Germany) and once with ACN. For protein digestion, Sequencing Grade Modified Trypsin (Promega, WI, USA) in 100 mM TEAB was used in an enzyme:protein ratio of 1:50 overnight at 37°C, which was stopped by addition of 100% ACN. Peptides were eluted in 2 fractions with 87% ACN in ammonium formate (Agilent Technologies, USA), followed by 2% dimethyl sulfoxide (v/v) (Sigma Aldrich, Germany). Fractions were evaporated to dryness and reconstituted in 0.1% (v/v) formic acid (Merck, Darmstadt, Germany) before subjection to LC-MS/MS analysis.

### LC-MS/MS

LC-MS/MS analysis of samples was performed on an UltiMate 3000 RSLCnano system (Dionex, Sunnyvale, CA, USA), online coupled to a Q Exactive HF mass spectrometer (Thermo Fisher Scientific, Waltham, MA, USA) by a chip-based electrospray ionization source (TriVersa NanoMate, Advion, Ithaca, NY, USA) as described before (85). Briefly, peptides were loaded on a trapping column (Acclaim PepMap 100 C18, 3 _m, nanoViper, 75 _m _ 5 cm, Thermo Fisher Scientific, Waltham, MA, USA) at a flow rate of 5µl/min using 2% ACN (v/v) and 0.05% trifluoroactitic acid (v/v). Peptides were separated on an analytical column (Acclaim PepMap 100 C18, 3 _m, nanoViper, 75 _m _ 25 cm, Thermo Fisher Scientific,Waltham, MA, USA) using a 150 min gradient of increasing ACN concentration (2-80%) in 0.1% formic acid.

### Proteomic data analyses

MS raw data were processed with MaxQuant Version 1.6.2.10 using the *Mus musculus* Uniprot reference proteome (reviewed and unreviewed entries). Default parameters were used with following exceptions: Carbamidomethylation of cysteine was set as fixed modification, whereas oxidation of methionine and acetylation of protein N-termini were set as variable modifications. Minimum peptide length was set to 7. Match between runs was activated. Proteins were quantified based on 2 unique peptides. MaxQuant quality control was carried out by PTXQC package^37^ in the R environment. Protein contaminants and reverse hits were excluded using Perseus 1.6.2.2. The data presented in the study are deposited in the ProteomeXchange Consortium via the PRIDE (86) partner repository, accession number PXD035883.

Protein intensities were log2-transformed, filtered for proteins quantified in at least 3 replicates per condition, followed by variance-stabilization and imputation using R’s DEP (87) package (fun = “MinProb”, q = 0.01) for proteins not quantified in any other replicates in the particular conditions. Furthermore, the following R packages were applied: limma (88), plyr (89), reshape2 (90), xlsx (91), ggsci (92), circlize (93), calibrate (94), ggplot2 (95), dendsort (96), readxl (97), qpcR (98), splitstackshape (99), tidyr (100), and Tmisc (101). Fold changes and p-values relative to the control were calculated using Student`s t-test. Proteins with a p-values of ≤0.05 were considered significantly regulated.

Alterations in biological pathway were determined using Ingenuity Pathway Analysis (IPA, Qiagen) using the mouse immune cell database. Pathways with a Benjamini-Hochberg corrected p-value (p.adj) ≤0.05 were considered significantly enriched. The DAVID Bioinformatics Resources 6.8 (102) were used to map Entrez Genes to Uniprot Accessions for supposedly interesting IPA pathways.

### Th17 polarization and adoptive T cell transfer into healthy pregnant mice

Naïve CD4^+^ T cells derived from the spleen and lymph nodes of virgin WT CBA/J females were cultured under Th17-differentiating conditions for 3 days in the presence or absence of 500 mIU/ml rhCG. Afterwards, total T_inf(Th17)_ cells were washed and adjusted to 1 × 10^6^ cells in 200 µl PBS (PAN Biotech). On gd 0–2, rhCG-treated or non-hCG-treated T_inf(Th17)_ cells were adoptively transferred into healthy pregnant BALB/c-mated WT CBA/J females *via* intravenous injection into the tail vein. Controls received 200 µl PBS only. All pregnant females were examined by high-frequency ultrasonography at different gds and were finally euthanized on gd 12.

### High-frequency ultrasonography

To follow intrauterine placental and fetal development, pregnant females underwent ultrasonographic examination at gd 8, 10 and 12 with the Vevo 2100 System (Fujifilm VisualSonics) as described previously (103). Briefly, female mice were anesthetized with isoflurane, fixed in dorsal position and ventrally depilated by cream application. Depending on the day of examination, implantation size (gd 8, 10 and 12), placental area, thickness and diameter (gd 10 and 12) were measured. Additionally, peak systolic velocities (PSVs) and EDVs of the UA were recorded and analyzed with the Vevo LAB software. The software automatically calculated RI = (PSV − EDV)/PSV and PI = (PSV − EDV)/velocity time integral.

### Determination of pregnancy outcome, tissue sampling and processing

On gd 12, each female was euthanized, the abdomen was opened and the bicornuate uterus was removed. Both uterine horns were opened longitudinally, and the implantation sites were collected. The number of total implantation and abortion sites was recorded. Abortion sites were identified by necrotic and hemorrhagic tissue residues, and the abortion rate was calculated as (number of abortion sites/total number of implantation sites) × 100. Fetoplacental units were separated from the surrounding tissue, and fetal and placental weights were measured with a microscale (Kern & Sohn). Spleen, lymph nodes (inguinal and para-aortic) and decidual tissue were collected and immediately transferred to ice-cold full RPMI 1640 medium or HBSS (ThermoFisher) supplemented with DTT (1mM, Sigma). Spleen and lymph nodes were forced through a 100 µm cell strainer (Corning) to obtain single-cell suspensions, which were exposed to erythrocyte lysis buffer, washed with full RPMI 1640 medium and then plated in multiwell plates at a density of 1 × 10^6^ cells/ml. Whole decidual tissue was cut into small pieces and incubated in DTT-containing HBSS. Dissociated cells were washed in HBSS, and lymphocytes were obtained by density gradient centrifugation, then plated in multiwell plates at densities of 3–5 x 10^5^ cells. All plated cells were stimulated with PMA (50 ng/ml), ionomycin (500 ng/ml) and brefeldin A (10 µg/ml) for 5 h, then analyzed by flow cytometry.

### Flow-cytometry analyses

Extracellular and intracellular Ab staining was performed to assess the purity of MACS-isolated T cells and to determine the frequencies of Th subsets and marker expression *in vitro* and *in vivo*. Briefly, cells were suspended in a flow-cytometry buffer consisting of PBS containing 1% bovine serum albumin (Merck Millipore) and 0.1% sodium azide (Sigma). Ab staining for extracellular markers was performed for 30 min at 4°C in the dark. All other cells, following a washing step in flow-cytometry buffer, were fixed for 1 h at room temperature in fixation buffer, then washed in permeabilization buffer and incubated in the presence of antibodies (diluted in permeabilization buffer) for 30 min at room temperature to achieve intracellular staining. Fixation, permeabilization and intracellular staining was performed according to the True-Nuclear™ Transcription Factor Staining Protocol provided along with the True Nuclear Transcription Buffer Set (BioLegend). The cells were washed again in permeabilization buffer, suspended in flow-cytometry buffer and analyzed with a multicolor flow Attune NxT Flow Cytometer (ThermoFisher). Data analysis was conducted with Attune NxT software. Primary gates were set on lymphocytes in FSC/SSC plots, followed by exclusion of doublets and dead cells. FMO controls were used to identify and gate cells in the context of fluorescence spread due to the multiple fluorochromes in a stained sample. Where necessary, individual gating strategies and FMO controls are provided within the figures. The following Abs were applied: AF700-labeled anti-mouse CD45 mAb (clone 30-F11, ThermoFisher), FITC-labeled anti-mouse CD4 mAb (clone RM4-5, BD Bioscience), eFluor506-labeled Fixable Viability Dye (FVD, 1:1000, ThermoFisher), PE-labeled anti-mouse CD25 mAb (clone 7D4, Miltenyi Biotec), APC-labeled CTLA-4 mAb (clone UC10-4F10-11, BD Bioscience), BV421-labeled anti-mouse PD-1 mAb (clone J43, BD Bioscience), PE-Cy7-labeled anti-mouse ICOS mAb (clone 7E.17G9, ThermoFisher), PE-labeled anti-mouse IL-17A mAb (clone TC11-18H10, BD Bioscience), APC-labeled anti-mouse RORγt mAb (clone AFKJS-9, ThermoFisher), BV421-labeled anti-mouse IL-10 mAb (clone JES5-16E3, BioLegend), PE-eFluor610-labeled anti-mouse FOXP3 mAb (clone FJK-16S, ThermoFisher), PerCP-Cy5.5-labeled anti-mouse TNF-α mAb (clone MP6-XT22, BD Bioscience), and PE-Cy7-labeled anti-mouse IL-2 mAb (clone JES6-5HU, BioLegend). All antibodies were diluted 1:200.

### Histological analysis of uSAs

One implantation per animal was fixed in 4% paraformaldehyde solution (Sigma) containing 0.1 M sucrose (Sigma) for 6 h, transferred into 70% ethanol and stored at 4°C overnight. The next day, the implantation was bisected; one half was further dehydrated in an ascending ethanol series, incubated in xylene (Carl Roth) and embedded in paraffin. Tissue sections (5 µm) transferred to glass slides were stained with hematoxylin–eosin to enable evaluation of uSAs. Three to eight uSAs in the decidua basalis of each implantation site were identified using an Axio Observer inverted microscope (Carl Zeiss). The outer circumference and lumen circumference of each uSA were measured at a magnification of 200×. From these measurements, the outer diameter and lumen diameter (circumference/π), wall-to-lumen ratio (outer diameter/lumen diameter) and the wall thickness ((outer diameter−lumen diameter)/2) were calculated.

### Data analysis and statistics

Data analysis was conducted with GraphPad Prism 7.0 (Statcon) and SPSS Statistics 24 (IBM) software. All datasets were analyzed for normal distribution using the Shapiro–Wilk test, to determine the use of parametric tests (normal distribution) or nonparametric tests (non-normal distribution). The test used for each dataset is indicated in the respective figure legend. In all cases, *p <*0.05 was considered to indicate a statistically significant difference between groups.

## Data availability statement

The data presented in the study are deposited in the ProteomeXchange Consortium via the PRIDE (86) partner repository, accession number PXD035883.

## Ethics statement

The animal study was reviewed and approved by Landesverwaltungsamt Sachsen-Anhalt (AZ42502-2-1515 UniMD).

## Author contributions

LL, ASt, NM, KS and IK performed and analyzed experiments. ASc and AZ designed and supervised experiments. LL and ASc prepared figures, interpreted data and wrote the manuscript. AZ and MB critically revised the manuscript. All authors contributed to the article and approved the submitted version.

## Funding

The present study was financially supported by a grant to ASc from the German Research Foundation (SCHU 2905/3-1) and intramural funding to AZ. LL and ASt were supported by a grant from the Medical Faculty of the Otto-von-Guericke University (Kommission zur Förderung des wissenschaftlichen Nachwuchses).

## Acknowledgments

KS, IK and MB thank the UFZ-funded ProMetheus platform for proteomics and metabolomics for the support of this project and Maj Schuster for excellent technical assistance.

## Conflict of interest

The authors declare that the research was conducted in the absence of any commercial or financial relationships that could be construed as a potential conflict of interest.

## Publisher’s note

All claims expressed in this article are solely those of the authors and do not necessarily represent those of their affiliated organizations, or those of the publisher, the editors and the reviewers. Any product that may be evaluated in this article, or claim that may be made by its manufacturer, is not guaranteed or endorsed by the publisher.
