# Supplementary material for: Human chorionic gonadotropin promotes murine Treg cells and restricts pregnancy-harmful proinflammatory Th17 responses
Source: Front Immunol. 2022 Sep 20;13:989247. doi: 10.3389/fimmu.2022.989247 (PMC9531259; doi:10.3389/fimmu.2022.989247)
Supplement: Supplementary file 1 [file DataSheet_1.docx]

Supplementary Material


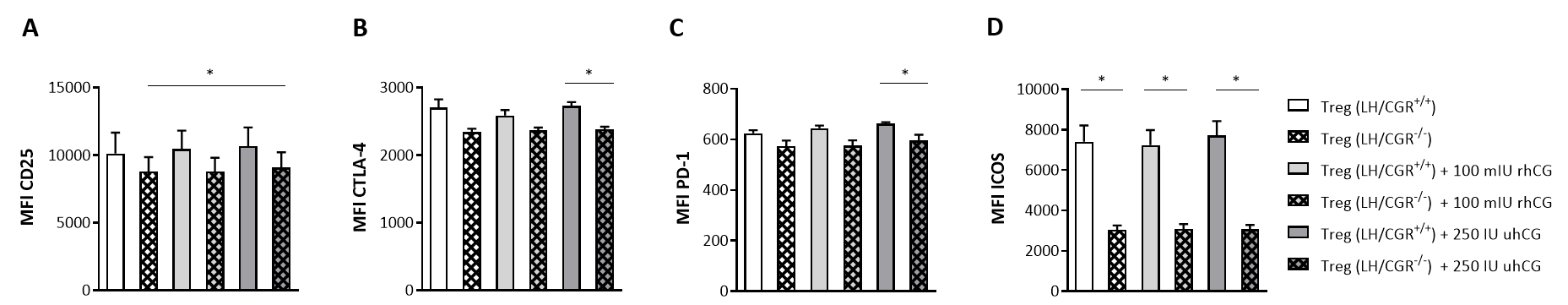


**Supplementary Figure 1** (A–D) Expression levels of CD25, CTLA-4, PD-1 and ICOS per CD4^+^ T cell as the mean fluorescence intensity (MFI). CD4^+^CD25^+^ T cells from wild-type (LH/CGR^+/+^; *n* = 6) or LH/CGR-deficient (LH/CGR^-/-^; *n* = 4) female mice were included in the assays. All assays were run in duplicate. Data are presented as the mean plus standard error of the mean (S.E.M.). Statistical analysis among LH/CGR^+/+^ or LH/CGR^-/-^ groups used the Friedman test followed by Dunn’s multiple-comparison test. For comparisons between WT and KO groups, the Kruskal–Wallis test was used, followed by Dunn’s multiple-comparison test. Following, means plus S.E.M. are provided for significant differences between LH/CGR^+/+^ plus uhCG vs LH/CGR^-/-^ plus uhCG for CTLA-4: 2730±55.35 vs 2381±41.89 and for PD-1: 662.1±5.861 vs 595.3±22.31; LH/CGR^+/+^ vs LH/CGR^-/-^ for ICOS: 7395±810.2 vs 3033±228.1 (w/o hCG), 7236±742.8 vs 3088±238.2 (rhCG), 7712±710.0 vs 3060±227.0 (uhCG) and LH/CGR^-/-^ w/o hCG vs LH/CGR^-/-^ uhCG for CD25: 8790±1067 vs 9083±1136 * indicates *p* <0.05. rhCG, recombinant hCG; uhCG, urine-derived hCG.


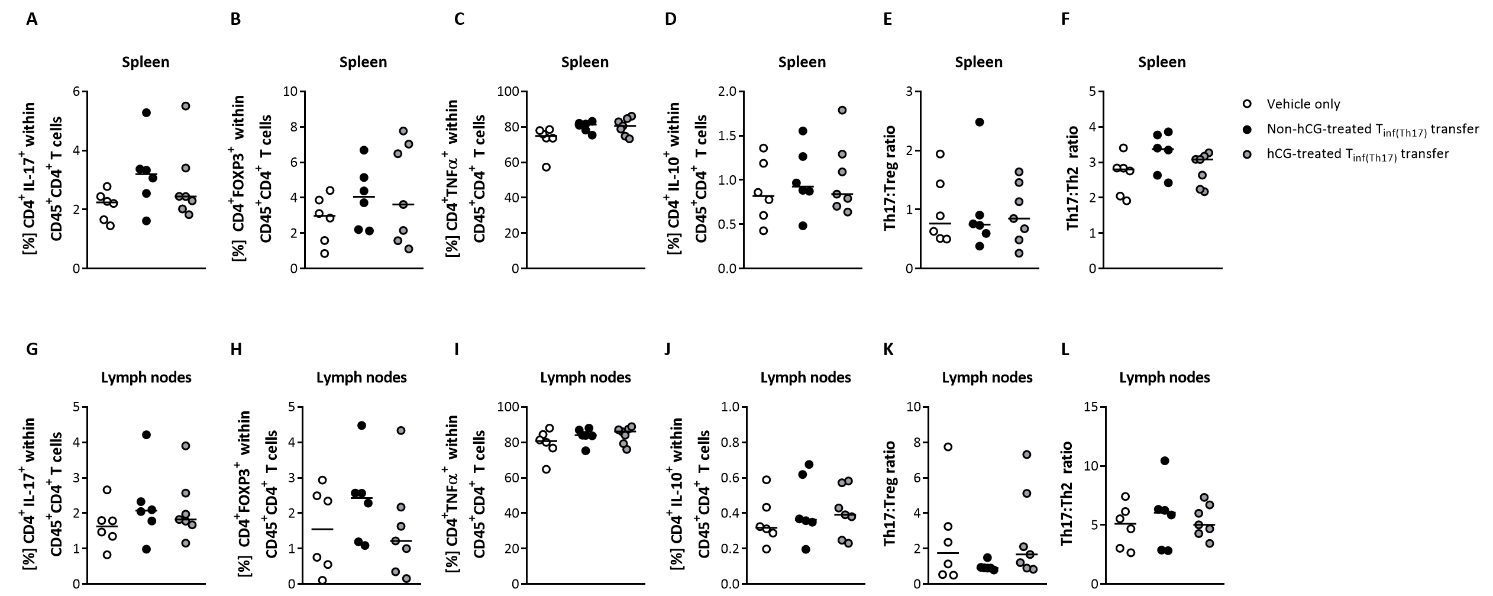


**Supplementary Figure 2** (A–F) Percentages of CD4^+^IL-17^+^, CD4^+^FOXP3^+^, CD4^+^TNFα^+^ and CD4^+^IL-10^+^ cells among CD45^+^ T cells, as well as Th17:Treg and Th17:Th2 ratios in cells from the spleen on gestational day (gd) 12. (G–F) Percentages of CD4^+^IL-17^+^, CD4^+^FOXP3^+^, CD4^+^TNFα^+^ and CD4^+^IL-10^+^ cells among CD45^+^ T cells, as well as Th17:Treg and Th17:Th2 ratios in cells from the lymph nodes (inguinal and para-aortic) on gd 12. Experimental groups were vehicle-only injection (*n* = 6), non-hCG-treated T_inf(Th17)_ transfer (*n* = 6), and hCG-treated T_inf(Th17)_ transfer (*n* = 7). Data are presented as medians showing individual values for each animal. For comparisons between experimental groups, the Kruskal–Wallis test was used, followed by Dunn’s multiple-comparison test. No statistically significant differences were identified.
